# The effectiveness of music therapy in improving behavioral symptoms among children with autism spectrum disorders: a systematic review and meta-analysis

**DOI:** 10.3389/fpsyt.2024.1511920

**Published:** 2025-01-14

**Authors:** Xiuyan Gao, Guangjun Xu, Ningning Fu, Qi Ben, Lin Wang, Xiumei Bu

**Affiliations:** ^1^ School of Nursing, Liaoning University of Traditional Chinese Medicine, Shenyang, Liaoning, China; ^2^ School of Health Management, Liaoyang Vocational College of Technology, Liaoyang, Liaoning, China

**Keywords:** music therapy, children, autism spectrum disorder, behavioral symptoms, meta-analysis

## Abstract

**Objectives:**

This comprehensive review and meta-analysis aimed to thoroughly identify the effectiveness of music therapy (MT) in improving behavioral symptoms in children with autism spectrum disorders (ASD) by analyzing the data from all relevant randomized controlled trials (RCTs) related to this field.

**Methods:**

From inception until September 18, 2024, PubMed, Web of Science, the Cochrane Library, SinoMed, and Embase were searched. Two reviewers extracted the data separately, and any controversies between the authors’ assessments were resolved by conversation or speaking with another author. The behavioral symptoms scale score before and after the intervention was taken from the included trials and used to reflect the therapeutic effect of music therapy in children with autism.

**Results:**

2607 records across all retrieved databases were discovered, thirteen of which were included in a meta-analysis with 1160 participants. According to the meta-analysis, children with autism showed a substantial improvement in their behavior symptoms when receiving music treatment (standardized mean difference [SMD] = -0.66, 95% confidence interval [CI]: -0.93 to -0.39, *p* < 0.001). With *I* ^2^ = 78% and *P* < 0.001, we did discover a medium level of heterogeneity among the included studies.

**Conclusions:**

MT has a positive impact on improving behavioral symptoms in children with autism. However, given the significant heterogeneity and limitations in this study, RCTs with rigorous methodological quality are still required to confirm the curative benefits of MT in autistic children precisely.

**Systematic review registration:**

https://www.crd.york.ac.uk/PROSPERO/, identifier CRD42024597939.

## 1 Introduction

Autism spectrum disorder is a persistent neurological upgrowth disorder characterized by difficulty in social contact, limited interests, and repetitious behaviors (1). In line with reports, throughout the previous 20 years in the United States, the frequency of autism spectrum disorder has varied from 2 in 10,000 to 1 in 54 (2). A study in 2019 by Sun et al. estimated that the incidence of autism spectrum disorder was approximately 1% in China (3), and the prevalence of boys was about four times that of girls (4). Additionally, an updated systematic review indicated that by 2021, the global average prevalence of autism was estimated to be about 1% (5). Not only that the number of children diagnosed with autism keep increasing, but children with autism may experience a range of abnormal behaviors in their early years, including resistance, irritability, social disengagement, stereotyped behavior, and incorrect speech when compared to children with typical development. This could explain why they can’t form positive peer and family relationships and lack the social skills necessary to adjust to normal social situations. Despite autism having significant negative effects on children and parents in the field of daily life, financial, physical, and mental health (6, 7), the effective treatments available to reduce the incidence of behavioral issues in children with autism are still few and untested. Thus, exploring scientific and efficient therapeutic measures to lessen the symptom severity of children with autism and improve behavioral symptoms is urgent.

As a result of the complex pathophysiology, which is linked to the interaction of early developmental environmental factors and genetics, there is an ongoing debate over the efficacy of current treatments for autism spectrum disorder in addressing behavioral symptoms. Applied behavioral analysis (8), cognitive behavioral therapy (9), sensory integration training (10), and medication therapy (11) are a few examples of interventions that have been used to address behavior problems in the past, but they always require a longer course of treatment, and because autistic children differ greatly from one another, the safety and efficacy of these therapies are not currently sufficiently supported by the available evidence (12). Thus, there is a pressing need to find novel cure therapy due to the increased prevalence of autism spectrum disorder and the lack of effective interventions for improving behavioral symptoms.

Drawing from the theory of behavioral, cognitive, and humanistic, an expanding body of randomized clinical research has explored the efficacy of music or music therapy in improving behavioral symptoms in children with autism spectrum disorder. Music therapy has been defined as “a systematic process of intervention that the eligible music therapist makes advantage of musical experience and the connections that foster through them as a dynamic process of transformation to improve patients’ health (13), while music intervention (e.g., music listening, music training, and singing, etc.) fails to make people relate to others, to communicate, and to share their feelings as a result of paying little attention on the intrinsic need of patients. Hence, music therapy, as a child-centered method, is gradually performed to support health and psychological development (14), where a qualified therapist concentrates on the children’s immersion, actions, and interests (15). Children with autism spectrum disorder would have their cortical and subcortical brain areas, which are linked to emotions and rewards, stimulated when receiving music therapy (16), which could help improve social motivation and emotional resonance in autism (17). Additionally, the mirror neuron system in the brain, which is beneficial to enhance imitation behavior will be strengthened in the engagement of music activities (18). As a cost-effective and non-invasive complementary adjunct therapy, music therapy shows its unique value in the treatment of autism spectrum disorder and makes children exhibit a strong preference for music (19). The effectiveness of music therapy in improving behavior symptoms (e.g., social interaction (20), general behavior, emotion sharing and recognition (21), imitation, and social skills (22), etc.) has been backed by certain studies conducted both at home and globally. However, the clinical therapeutic benefits of music therapy were called into question when a recent multi-center randomized controlled trial found that no statistically dramatic difference between music therapy groups and enhancement standard treatment groups was detected from the outlook of improving social effects before and after intervention (23). As a result, the efficacy evidence to support that music therapy has positive therapeutic benefits for children with autism spectrum disorder is still untenable.

Notably, a thorough evaluation of the literature revealed a dearth of research-based music therapy programs for reducing the behavioral symptoms of autism in kids. The bulk of small sample size randomized clinical trials, brief intervention length, inadequate methodological design, and insufficient follow-up highlight the need for additional research on the efficacy of music therapy in addressing behavior issues among those with autism spectrum disorder. Even though a few scholars have carried out meta-analyses regarding the efficacy of music therapy in the cure of autism spectrum disorder (24, 25), in contrast to earlier studies, this meta-analysis attempts to impartially evaluate the available data about the effectiveness of music therapy in reducing the behavioral symptoms in children with autism spectrum disorder to offer new perspectives and approaches to lessen the likelihood of behavioral abnormalities in this population.

## 2 Methods

This meta-analysis, registered on PROSPERO (CRD42024597939), was conducted according to the updated Preferred Reporting Items for Systematic Reviews and Meta-Analysis statement (26).

### 2.1 Search strategy

Two authors independently conducted a comprehensive retrieval of the databases of the Cochrane Library, SinoMed, PubMed Embase, and Web of Science from inception to September 18, 2024, to acquire the most knowledge in this field of research as well as because of the fewer studies about MT in ASD in the past. We utilized the search terms with medical subject headings or a combination of free text words and concepts related to children with ASD and MT, such as (“autistic disorder” or “autis* spectrum disorders” or “early infantile autism” or autis* or “autistic traits” or ASD), AND (“music therapy” or “music training” or “music intervention” or improvis* or music), without regard to language or status of the publication. Scanning was also carried out of the references in the contained studies manually to identify studies that we may have missed (see **Supplementary Table S1**).

### 2.2 Inclusion and exclusion criteria

PICOS (Population, Intervention, Comparator, Outcomes, Study Design) criteria were used to select studies, as outlined in **Table 1**.

**Table 1:** PICOS criteria for inclusion of studies.

Parameter	Criteria
Population	Children and adolescents under 18 years old and were diagnosed with ASD
Intervention	Studies involving MT performed by a trained therapist
Comparator	studies with no limitations on control measures
Outcomes	Studies reporting behavioral ability associated with ASD
Study design	Studies with randomized controlled trials in peer-reviewed journals with a Journal Citation Reports Index

ASD, autism spectrum disorder; MT, music therapy.

Criteria used for inclusion: (P) kids and teenagers under the age of eighteen were diagnosed with ASD as defined in DSM-5 (27) or ICD-11 (28) criteria; (I) involving music therapy (e.g., Mozart music, Orff music, Chinese medicine Wuxing-music, improvisational music therapy, etc.) delivered by a professional music therapist in the experiment group; (C) studies without limitations on comparison (no treatment or standard care); (O) employing appropriate measurement tools to appraise behavioral symptoms and providing clear data on behavioral symptoms in change score for easily evidence synthesis; (S) only randomized controlled trials were included, which were deemed to represent the higher quality of evidence, but studies with the design of single-case experimental were excluded.

Criteria used for exclusion: studies relating to animal experiment and intervention research; unavailable or unclear data after contacting the authors of studies; descriptive reviews or systematic reviews; books, study protocols, and letters from conferences; case study.

### 2.3 Literature screening and data extraction

All of the records that were searched were entered into EndNote X9, a reference management program. After removing duplicate entries, two writers (X. Gao and Q. Ben) independently assessed the recovered studies based on their evaluation of the abstracts and titles to identify potentially eligible studies. Following their reading of the entire material that appeared to be eligible, the two authors then went on to evaluate individual studies to determine which study was eventually included in further analysis. The other two writers, X. Gao, and N. Fu, used a formal sheet to capture information from each study on the first author’s name, the country of study, the year of publication, the study design, sample size, age, recommended interventions, behavioral symptoms linked to ASD, etc. Any discrepancies were resolved by discussion of screening criteria, otherwise, a third author (X. Bu) was consulted and made arbitration on the disagreements.

### 2.4 Study quality assessment

We carried out a thorough quality evaluation based on the Cochrane risk-of-bias instrument for randomized trials by two authors (X. Gao and N. Fu) separately to determine the possible influence of bias in the assessed studies. The exact components considered in this assessment were the following: random sequence creation, hidden allocation, blinding of participants and staff, outcome assessment blinding, inadequate outcome data, selective reporting, and other biases (29). Three categories were used to classify each bias: high danger, unknown danger, and low danger following the sheet with details on the available information that led to each judgment. Any disagreements among the authors’ assessments were resolved by consultation or assistance from the final author (X. Bu).

### 2.5 Statistical methods

Review Manager (V 5.4) assessed the bias risk for the randomized controlled trials, and all meta-analyses and realizations were valued based on STATA (V 15.0) and Review Manager (V 5.4). Standard mean difference and its 95% confidence interval were chosen as the effect size as all of the outcome indicators were continuous variables and distinct measurement tools were employed for the same intervention outcome indicator. In addition, SMD values of 0.2-0.49, 0.5-0.8, and > 0.8 were deemed to represent small, medium, and large differences, respectively (30). To quantify the heterogeneity among the literature, the *p*-value of Cochran’s Q test and the *I* ^2^ test, which is advised by Cochrane Reviews and gives an estimate of the proportion of variance of effect sizes that results from clinical and methodological heterogeneity rather than chance were used (31). Values of 50%, 50-75%, and 75% indicate small, moderate, and high levels of heterogeneity, respectively (32). When there was no substantial heterogeneity *(p* ≥ 0.1 and *I* ^2^ ≤ 50%), the fixed effects model was employed for the meta-analysis; in other cases, the DerSimonian and Laird random effects model, which considers variance between and within studies (33), and the source of heterogeneity were examined (32).

In the subgroup and meta-regression analysis (at least ten studies included), which were conducted to look at underlying factors impacting effect size, the following factors were found to be moderators: publication year, sample size, music style, the modality of intervention, measuring methods, and time of intervention. The results of the meta-analysis were verified for reliability and robustness using a leave-one-out sensitivity test. In addition, we examined publication bias using Egger’s test (34) and funnel plots. If *p* < 0.05 and the publication bias tests’ funnel plot failed to show a harmonious symmetry, we further used the trim-and-fill method (35), which recalculates the pooled SMD to incorporate hypothetical missing studies as needed, to account for the predicted publication bias.

## 3 Results

### 3.1 Results of the literature search

**Figure 1:**
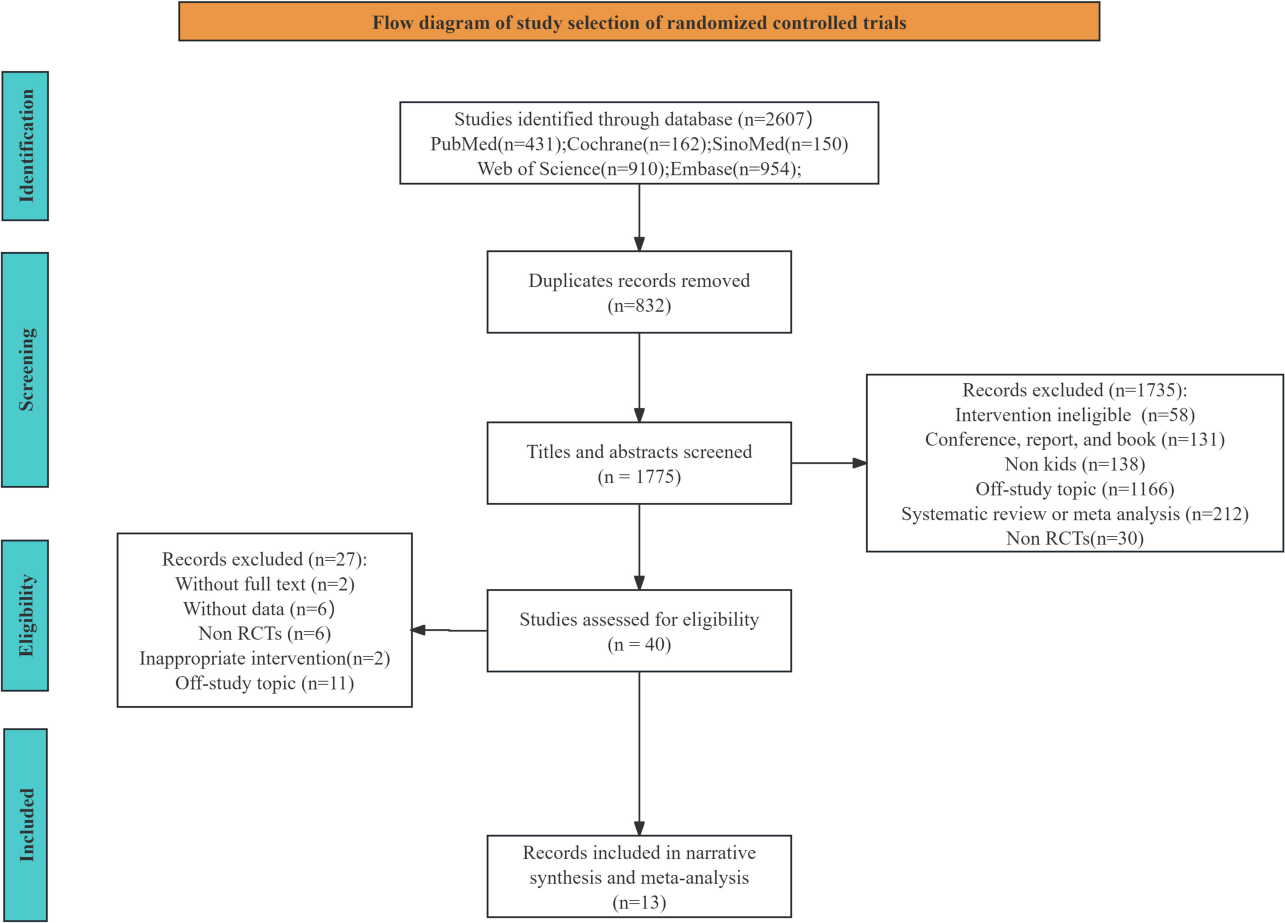
PRISMA flow diagram for the inclusion and selection of research.

The process for screening and choosing literature is depicted in **Figure 1**. Five electronic databases produced 2607 results for the first search. Just forty studies were reviewed in full after duplicates were removed and titles and abstracts were carefully examined. Six of the forty-three research had unclear study data, while two of the studies had incomplete text availability. There were only thirteen studies included in the systematic review and meta-analyses after we found that nineteen of the studies were unsuitable because of the study topic, study design, and intervention measures.

### 3.2 General characteristics of included literature

**Table 2** lists the essential details of the thirteen included studies, promoting clarity and making it easier to comprehend the main traits of the studies that were part of our analysis. Thirteen studies with 1160 participants were included in this systematic review and meta-analysis. Only three studies (16, 25, 41) did not report data of equal sample sizes based on the data analyzed during pre- and post-music therapy intervention. Except for the study by Yurteri N et al. (38), all studies included boys and girls with ages ranging from one to fourteen years. All studies used music therapy in different music styles, such as Orff music therapy (36, 41), Chinese medicine Wuxing-music (37, 39), Parent-child cooperative music therapy (43, 44), and singing bowls music therapy (45), etc. The studies in the analysis had intervention periods ranging from eight weeks (38) to one year (46). Of these, seven studies conducted interventions time twelve weeks or longer, while the remaining six studies all had interventions lasting ten weeks or less. Regarding the measurement instruments, only one study (25) utilized an autism diagnostic observation schedule (ADOS) and the remaining studies all used an autistic behavior checklist (ABC).

**Table 2:** Basic characteristics of literature.

Study	Year	Country	Study design	Age (Year)	Sample size	Intervention	Control	Intervention prescriptions	Behavioral symptoms (scale)
Bieleninik, L (25)	2021	Multi-center Nine countries	RCT	4-7	314	N=165 enhanced standard care + IMT	N=149 Enhanced standard care	One-to-one; 30 minutes three times a week for 20 weeks or 30 minutes once a week for 20 week	ADOS
Fan, Q L (33)	2024	China	RCT	3-6	93	N=48 comprehensive rehabilitation + Orff music therapy	N=45 Comprehensive rehabilitation	Group; 40 minutes twice a week for 24 weeks	ABC
Rabeyron, T (16)	2020	France	RCT	4-7	36	N=19 MT	N=17 ML	Group; 30 minutes once a week for 32 weeks	ABC
Yurteri, N (32)	2019	America	RCT	2-7	24	N=12 IMT	N=12 Usual care	Group; 40 minutes twice a week for 8 weeks;	ABC
He, Y X (36)	2022	China	RCT	3-5	100	N=50 Parent-child cooperative music therapy + ABA	N=50 ABA	Group (Offline); Online: 5-10 minutes once a week for 8 weeks with Mozart music; Offline: 60 minutes once a week for 8weeks with Orff music;	ABC
Li, N (37)	2021	China	RCT	2-7	60	N=30 Usual care + singing bowls music therapy	N=30 Usual care	Group; 30 minutes three times a week for 24 weeks; Treatment 10 times and rest 20 days	ABC
Rao, J M (35)	2023	China	RCT	6-8	102	N=51 Usual care + Chinese medicine Wuxing-music	N=51 Usual care	One-to-one and Group; 30-35 minutes six times a week for 12 weeks	ABC
Shen, S (38)	2022	China	RCT	2-9	75	N=38 Usual care + Parent-child cooperative music therapy	N=37 Usual care	Group (Offline); Online: 5-10 minutes once a week for 24 weeks; Offline: 60 minutes once a week for 24 weeks	ABC
Xiao, Q (39)	2023	China	RCT	3-9	80	N=40 Auditory integrated training + MT	N=40 Auditory integrated training	Group; 40 minutes five times a week for one year;	ABC
Zhang, L (34)	2023	China	RCT	3-7	80	N=40 Repetitive transcranial magnetic stimulation + Orff music therapy	N=40 Repetitive transcranial magnetic stimulation	Group; 20 to 30 minutes every 3 days for 12 weeks;	ABC
Zhao, Y H (40)	2020	China	RCT	≤14	68	N=38 Usual care + MT	N=30 Usual care	Group; 60 minutes two times a week for 15 weeks	ABC
Zhou, Y (41)	2023	China	RCT	1-6	32	N=16 Usual care + Chinese medicine Wuxing-music	N=16 Usual care	One-to-one and group; 12 weeks	ABC
Zhou, Y H (42)	2018	China	RCT	1-7	96	N=48 MT	N=48 Speech training	Group; Two times a week for ten weeks	ABC

MT, Music Therapy; IMT, Improvisational Music Therapy; ML, Music Listening; ABC, Aberrant Behavior Checklist; ADOS, Autism Diagnostic Observation Schedule; ABA, Applied Behavior Analysis.

### 3.3 Risk of bias

**Figure 2:**
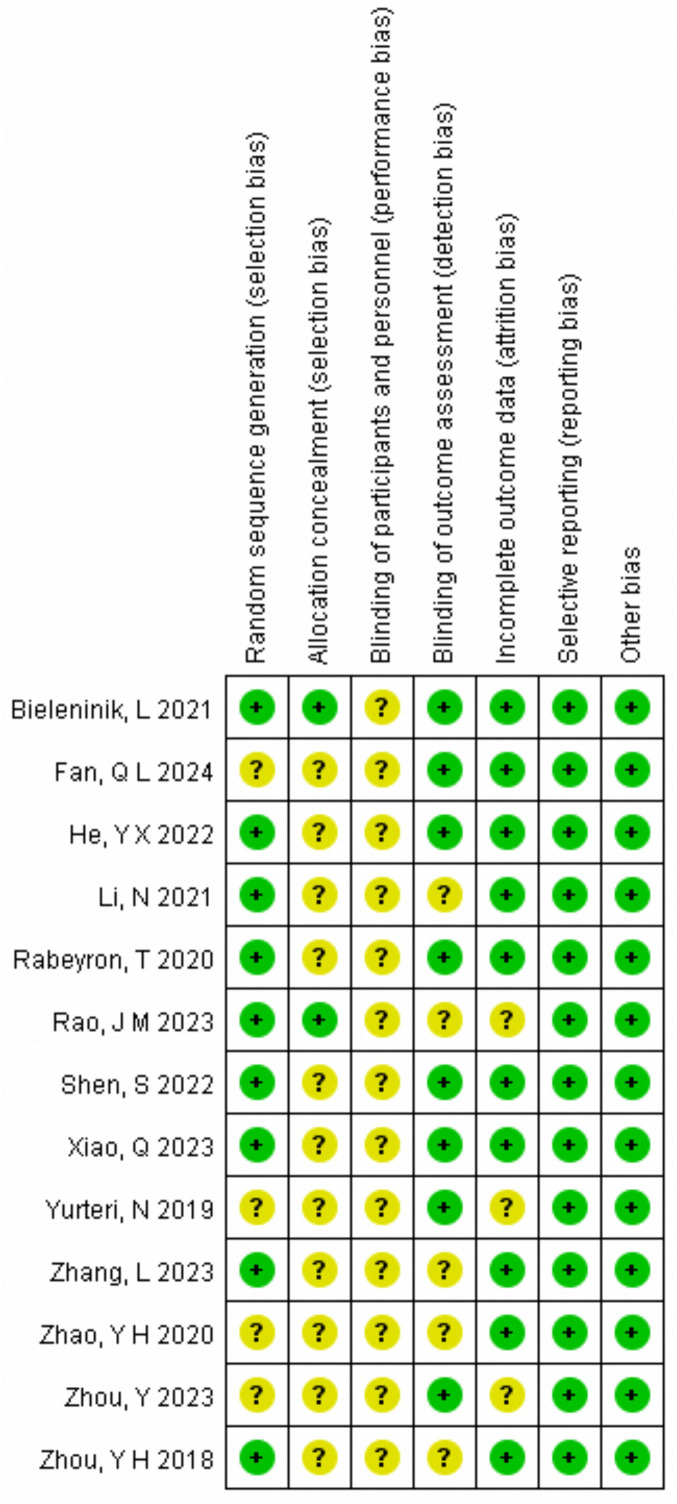
Assessment of risk of bias in included studies.

An overview of the risk of bias findings targeting every eligible literature is given in **Figure 2**. The PRISMA guidelines were followed in the evaluation of the quality of the available evidence. There was little chance of reporting bias and other biases in any of the 13 studies. It is challenging to keep participants unaware that they are taking part in a music therapy intervention, and none of the included research met the high standards required for a double-blind, randomized controlled study design. Just two studies (25, 37) revealed allocation concealment, but the majority of included research (n = 9) were rated as low risk in random sequence generation. Consequently, the evaluated literature was deemed to be of “moderate” quality in general.

### 3.4 Effectiveness of music therapy on behavioral symptoms

**Figure 3:**
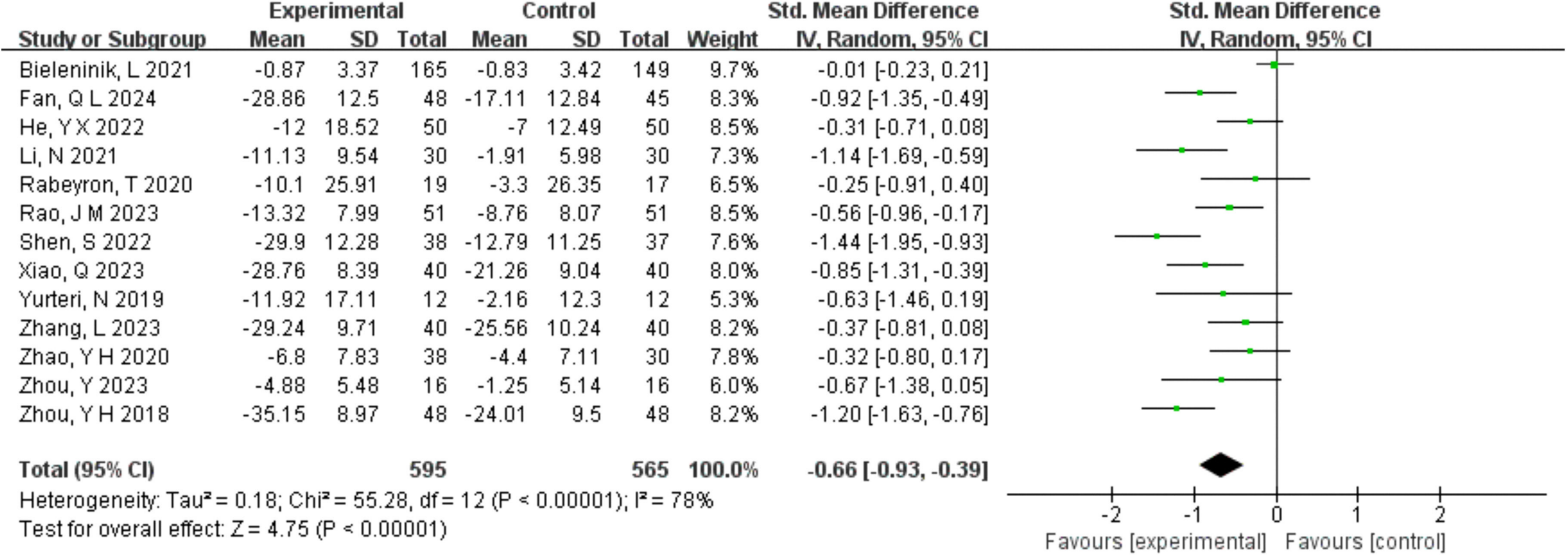
Meta-analysis of the effectiveness of music therapy on behavioral symptoms.

Thirteen research evaluated the impact of music therapy on the behavioral symptoms in kids with autism spectrum disorder. Of these 1160 participants, one study (25) used the ADOS scale to assess the effectiveness of music therapy on children with autism spectrum disorder, and the results showed no significant difference in improving behavioral symptoms between the music therapy group and the control group. The other studies, on the other hand, all used the ABC scale, and a significant difference in the overall improvement of behavioral symptoms related to autism spectrum disorder was found. A random effects meta-analysis of 13 randomized controlled studies was conducted, taking into account maximum heterogeneity (*p* < 0.05, *I* ^2^ = 78%). The results indicated a significant overall effect size (pooled SMD = -0.66, 95% CI: -0.93 to -0.39, Z = 4.75, *p* < 0.001) in improving behavioral symptoms in autistic children in comparison to the control group. The combined analysis results are displayed in **Figure 3**.

### 3.5 Meta-regression and subgroup analysis

The analysis of randomized controlled studies yielded an *I* ^2^ estimate of inter-study heterogeneity of 78% on account of the differences in sample size, demographics, and characteristics between music therapy groups and control groups. Thus, to determine the factors that influence heterogeneity among the included research, a variety of potential variables were coded and examined. For publication year, sample size, music style, intervention type, measurement tools, and intervention duration, a preliminary meta-regression analysis was conducted in the set of 13 randomized controlled studies. The results showed that none of the factors’ moderating effects were statistically significant (see **Supplementary Figure S2**).

Subgroup analyses on measurement tools, intervention duration, sample size, intervention type, publication year, and music type were further performed to confirm whether some factors may alter the results of meta-analysis and explore the influencing factors of heterogeneity. Surprisingly, the results of subgroup analysis revealed that no statistically significant variation was found in the amelioration of behavioral symptoms in children with autism between the experiment and comparison group in the subgroup of ADOS (pooled SMD: -0.01, 95% CI: -0.23-0.21, *p* = 0.92), sample size≥100 (pooled SMD: -0.26, 95% CI: -0.60-0.08, *P* = 0.13), one-to-one intervention (pooled SMD: -0.35, 95% CI: -0.81-0.11, *p* = 0.13), and parent-child cooperative music therapy (pooled SMD: -0.86, 95% CI: -1.96-0.24, *P* = 0.12). In addition, the behavioral symptoms scores were higher in trials with intervention durations of < 12 weeks than in those with ≥ 12 weeks (SMD = -0.69 vs. -0.61) and studies published before and after 2020 did not significantly differ concerning the publication year (see **Supplementary Figures S3A, B**). However, the outcomes of all subgroup analyses did not identify any potential influencing factors of heterogeneity.

### 3.6 Sensitivity analysis and publication bias

To verify the validity of the overall meta-analysis results and ascertain the source of heterogeneity, we conducted a sensitivity analysis on the included papers. Upon eliminating any specific study, the overall effect size stayed mostly unaltered. As a result, the meta-analysis’s conclusions were generally trustworthy and solid (see **Supplementary Figure S4**). Surprisingly, we also discovered that the removal of this study (17) significantly reduced overall heterogeneity, with an *I* ^2^ of 60% and a *p*-value of 0.004, suggesting that this study may be responsible for the observed heterogeneity. We have chosen to investigate and debate it further because it presented additional challenges for the ongoing application of music therapy. This study was a global clinical investigation that was not restricted to any particular set of music intervention techniques or music therapists, which added difficulties in conducting persistent implementation of music therapy. Alternatively, a notable distinction in methodology, such as selecting a proximal or distal outcome could account for the variation.

The main purpose of the funnel plot was to detect any publication bias among the included studies. There was a dissymmetry in the funnel plot result among the thirteen included papers, indicating a higher probability of publication bias. Then Egger’s test was further used to confirm the probability of publication bias. The trim-and-fill analysis revealed that although publication bias was also found in Egger’s tests (*p* = 0.029), it had no bearing on the total effect size (see **Supplementary Figures S5A–C**).

## 4 Discussion

In contrast to earlier research, our meta-analysis was the first study to explore the effects of music therapy focusing on the behavioral symptoms in children diagnosed with autism spectrum disorder. Though we expanded the scope of the literature review by retrieving a range of studies employing music therapy, including singing bowls music therapy, parent-child cooperative music therapy, Chinese medicine Wuxing-music, and Orff music therapy, only 13 randomized clinical studies, with a moderate risk of bias were included in this meta-analysis, and the total sample consisted of 1160 patients who were 14 years of age or younger. By combining the results of all the studies, we could conclude that either using music therapy exclusively or in conjunction with conventional treatment, children with autism may indeed see improvements in their behavioral symptoms. However, due to the differences in methodology and clinical, such as music style, duration of the intervention, assessment tools, the choice criteria of the music therapist, age, and severity degree of autism spectrum disorder, among other factors, the *I* ^2^ value for the behavioral symptoms was 78%, indicating significant heterogeneity between included studies. Despite sensitivity analysis, funnel plots, and other methods detecting publication bias were applied to verify the robustness and dependability of the combined effect result and publication bias, respectively, which indicated that there was no publication bias and the meta-analysis result was generally robust and dependable, we did not exactly discover the possible source and affecting factors of heterogeneity by further detecting and analyzing meta-regression and subgroup analysis, as first reported. Therefore, it is urgent that much strict RCTs are required to further confirm the evidence and provide more comprehensive insights into the therapeutic effects of music therapy on behavioral symptoms in children with autism.

In the meta-analysis, the pooled SMD (-0.66, 95% CI: -0.93 to -0.39, Z = 4.75, *p* < 0.001) showed music therapy indeed improves the ASD-related behavioral symptoms, which is consistent with the earlier studies (20, 47). Over the past few years, research on the therapeutic benefits of music therapy for children with autism spectrum disorder has been conducted. Children with autism respond favorably to music for its safe and controlled stimulus (25), which allows children with autism to participate in music experience positively and promotes the improvement of social interaction and social skills in turn (48). Additionally, Music therapy, as a child-centered intervention measure, can enhance their sense of participation in the training of child’s social communicative skills (15), which probably helps to improve quality of life and reduce the level of symptom severity of children with autism (24). Because of its great adaptability and flexibility, Kemper et al. suggested that music therapy, as a non-drug and supplemental treatment, has potential benefits in the development of physiology and psychology (49). When it comes to music therapy’s ability to help autistic children with their behavioral issues, it does so by stimulating the brain’s sensory system with its rhythm, melody, and harmony, which creates pleasant and dependable sensory experiences that lessen the occurrence of maladaptive behaviors (50). Furthermore, music can activate brain networks to maximize target behaviors through synchronized neuronal firings when children with autism are exposed to similar musical and nonmusical tasks (25). However, the comprehensive multicenter randomized clinical study by Bieleninik, L (25) did not show a significant statistical difference between the improvisational music therapy group and control group in terms of symptom severity (e.g., social affect and social responsiveness) after 20 weeks of intervention. Therefore, more investigation is needed to confirm the effectiveness of music therapy in the improvement of behavioral symptoms in children with autism.

As before shown, the combined results of studies investigating the association between music therapy and behavioral symptoms indicated that music therapy had positive impacts on improving behavioral symptoms in children with autism. However, the results of subgroup analysis in the measurement tools showed that there was no statistical significance identified in the use of ADOS between two groups in the improvement of behavioral symptoms in children with autism. The ADOS, as a standardized tool for medical diagnosis of autism, has been shown to have significant effectiveness in the classification of autism, but it is less sometimes sensitive for distinguishing children with mild autism (51). Similarly, the study by Rabeyron, T. et al. found that the ABC scale encompassing larger constructs of physical health, impatience, and hyperactivity did not show any overall improvements in behavioral problems. Surprisingly, the lethargy and stereotypy subscale scores showed a significant difference between the music therapy group and the music listening group (26), which could be explained by the fact that vestibular movements, a natural response to music therapy (52), would enhance lethargy behavior by facilitating motor skills. Hence, a possible hypothesis could be concluded that for measurement instruments, different features of the scale or subscale and their unique sensitivity to outcome change may result in inconsistent results, even if subgroup analysis in this study did not reveal a statistically significant difference between the ABC and ADOS scales. Therefore, when conducting relevant studies, it is necessary to choose the appropriate measurement tools based on the aim of the study instead of excessively global behavioral symptoms composite scores to explore the effectiveness of music therapy on behavioral symptoms in children with autism.

Regarding the music type, though the subgroup analysis found that parent-child cooperative music therapy did not show significant improvement in behavioral symptoms in children with autism, Redondo et al. reported Orff music could effectively help improve repetitive behavior problems (53). A probable reason might be that associated brain regions to govern sympathetic nerves will be activated in the role of Orff music therapy, and then promote the unleash of neurotransmitters. We further discovered that the key factors contributing to the dramatical differences were the context of music therapy implementation, intervention personnel of music therapy, and methodology difference in sample size, intervention duration, and intervention intensity, etc., which could also explain that why the study by Bieleninik, L, et al. did not find any statistically significant difference of improvisational music therapy in improving symptom severity in children with autism (25). Regarding the length of intervention, the subgroup analysis showed that ≤ 12 weeks intervention period significantly reduced the associated behavioral issues in children with autism spectrum disorder (pooled SMD = -0.61, 95% CI: -0.90 to -0.32, *p* < 0.001). Furthermore, the ≤ 12 weeks intervention period showed a more significant improvement in the associated behavioral issues in children with autism spectrum disorder than the > 12 weeks intervention period (pooled SMD = 0.69, 95% CI: -1.14 to -0.25, *p* = 0.002). Remarkably, Shi, Z. et al.’s study (54) suggested that the intervention should last for at least 12 weeks. Nonetheless, the beneficial effects of music therapy persisted across a shorter time frame of less than 12 weeks (47). Perspectives arguing much advantages with longer music therapy intervention in children with autism were not confirmed by randomized trials with high quality, even though varied durations of music therapy usually produce different therapeutic outcomes. Thus, future research should follow the child’s actual clinical situation (e.g., preference, behaviors, and the severity degree of autism, etc.) and the research goal to choose the reasonable music type and intervention length.

To sum up, standard music therapy prescriptions have not yet reached a consensus, indicating that different methodology designs (intervention duration, measurement instrument, criteria of the music therapist and music style, etc.) and clinical situations should be seriously considered. To some extent, music therapy may indeed improve behavioral symptoms in children with autism spectrum disorder. However, rigorous random controlled studies testing the curative effects of music therapy on behavioral symptoms in children with autism remain necessary.

## 5 Limitations

In addition to covering a larger body of research, this meta-analysis addressed publication bias, sensitivity analyses, and more thorough subgroup analyses. As such, our research may contribute to a better comprehension of the therapeutic benefits of music therapy for children diagnosed with autism spectrum disorder. Our study did, however, still have a few potential flaws. Firstly, the quantity of studies involved may be less than the general amount of acceptable research because only five databases were examined. Secondly, the limited number of studies, and the most of studies were conducted in China included in this meta-analysis precluded a thorough investigation of the source of heterogeneity. Third, the validity of our findings may have been diminished by the small sample sizes and the restricted number of studies conducted. Finally, given the majority of the included studies did not indicate adverse events, it was difficult for us to generate a trustworthy evaluation of the security of music therapy for children with autism spectrum disorder.

## 6 Conclusions

In summary, the meta-analysis’s findings confirmed the therapeutic effectiveness of music therapy for reducing behavioral symptoms in kids with autism spectrum disorder. The evidence supporting the effectiveness of music therapy in improving behavioral symptoms in children with autism spectrum disorders was still insufficient because of the restricted sample size, low design quality, and the condition that the majority of the included studies were conducted in China. Meanwhile, the conclusions regarding the therapeutic effectiveness of music therapy in continuous intervention should be handled cautiously due to the notable methodological and clinical variability across the included research. Subgroup analysis showed that the therapeutical effectiveness of music therapy in improving behavioral symptoms among children with autism spectrum disorder did not increase with longer intervention times, which contradicted earlier research on the best period of intervention for autistic children. Therefore, rigorous adherence to trial criteria is required in the conduct of randomized, assessor-blind, multi-center-controlled research to arrive at a consensus regarding the long-term effectiveness of music therapy. To provide more precise and reliable data, researchers should take into account the severity of autism in children as well as the mechanism underpinning music treatment.

## Data Availability

The original contributions presented in the study are included in the article/**Supplementary Material**. Further inquiries can be directed to the corresponding author.

## References

[1] Battle DE . Diagnostic and statistical manual of mental disorders (DSM). Codas. (2013) 25:191–2. doi: 10.1590/s2317-17822013000200017 24413388

[2] Maenner MJ Shaw KA Bakian AV Bilder DA Durkin MS Esler A . Prevalence and characteristics of autism spectrum disorder among children aged 8 years - autism and developmental disabilities monitoring network, 11 sites, United States, 2018. MMWR Surveill Summ. (2021) 70:1–16. doi: 10.15585/mmwr.ss7011a1 PMC8639024 34855725

[3] Sun X Allison C Wei L Matthews FE Auyeung B Wu YY . Autism prevalence in China is comparable to Western prevalence. Mol Autism. (2019) 10:7. doi: 10.1186/s13229-018-0246-0 30858963 PMC6394100

[4] Lord C Elsabbagh M Baird G Veenstra-Vanderweele J . Autism spectrum disorder. Lancet. (2018) 392:508–20. doi: 10.1016/S0140-6736(18)31129-2 PMC7398158 30078460

[5] Zeidan J Fombonne E Scorah J Ibrahim A Durkin SM Saxena S . Global prevalence of autism: A systematic review update. Autism Res. (2022) 15:778–90. doi: 10.1002/aur.2696 PMC9310578 35238171

[6] Hossain MM Khan N Sultana A Ma P McKyer ELJ Ahmed HU . Prevalence of comorbid psychiatric disorders among people with autism spectrum disorder: An umbrella review of systematic reviews and meta-analyses. Psychiatry Res. (2020) 287:112922. doi: 10.1016/j.psychres.2020.112922 32203749

[7] Leigh JP Du J . Brief report: forecasting the economic burden of autism in 2015 and 2025 in the United States. J Autism Dev Disord. (2015) 45:4135–9. doi: 10.1007/s10803-015-2521-7 26183723

[8] Voss C Schwartz J Daniels J Kline A Haber N Washington P . Effect of wearable digital intervention for improving socialization in children with autism spectrum disorder: A randomized clinical trial. JAMA Pediatr. (2019) 173:446–54. doi: 10.1001/jamapediatrics.2019.0285 PMC6503634 30907929

[9] Wood JJ Kendall PC Wood KS Kerns CM Seltzer M Small BJ . Cognitive behavioral treatments for anxiety in children with autism spectrum disorder: A randomized clinical trial. JAMA Psychiatry. (2020) 77:474–83. doi: 10.1001/jamapsychiatry.2019.4160 PMC6902190 31755906

[10] Randell E Wright M Milosevic S Gillespie D Brookes-Howell L Busse-Morris M . Sensory integration therapy for children with autism and sensory processing difficulties: the SenITA RCT. Health Technol Assess. (2022) 26:1–140. doi: 10.3310/TQGE0020 35766242

[11] McCracken JT McGough J Shah B Cronin J Hong D Aman GM . Risperidone in children with autism and serious behavioral problems. N Engl J Med. (2002) 347:314–21. doi: 10.1056/NEJMoa013171 12151468

[12] Sharma SR Gonda X Tarazi FI . Autism Spectrum Disorder: Classification, diagnosis and therapy. Pharmacol Ther. (2018) 190:91–104. doi: 10.1016/j.pharmthera.2018.05.007 29763648

[13] Bruscia KE . Defining Music Therapy. 2nd edition. Gilsum (NH: Barcelona Publishers (1998).

[14] Rabeyron T Robledo Del Canto JP Carasco E Bisson V Bodeau N Vrait FX . A randomized controlled trial of 25 sessions comparing music therapy and music listening for children with autism spectrum disorder. Psychiatry Res. (2020) 293:113377. doi: 10.1016/j.psychres.2020.113377 32798927

[15] Geretsegger M Holck U Carpente JA Elefant C Kim J Gold C . Common characteristics of improvisational approaches in music therapy for children with autism spectrum disorder: developing treatment guidelines. J Music Ther. (2015) 52:258–81. doi: 10.1093/jmt/thv005 26019303

[16] Caria A Venuti P de Falco S . Functional and dysfunctional brain circuits underlying emotional processing of music in autism spectrum disorders. Cereb Cortex. (2011) 21:2838–49. doi: 10.1093/cercor/bhr084 21527791

[17] Mandic-Maravic V Grujicic R Milutinovic L Munjiza-Jovanovic A Pejovic-Milovancevic M . Dopamine in autism spectrum disorders-focus on D2/D3 partial agonists and their possible use in treatment. Front Psychiatry. (2021) 12:787097. doi: 10.3389/fpsyt.2021.787097 35185637 PMC8850940

[18] Pavlenko VB Kaida A Klinkov VN Mikhailova AA Orekhova LS Portugalskaya AA . Features of reactivity of the EEG MU rhythm in children with autism spectrum disorders IN helping behavior situations. Bull Russian State Med University. (2023), 24–30. doi: 10.24075/brsmu.2023.009

[19] Kanner L . Autistic disturbances of affective contact. Acta Paedopsychiatr. (1968) 35:100–36. doi: 10.1111/j.1651-2227.1968.tb06978.x 4880460

[20] Geretsegger M Elefant C Mössler KA Gold C . Music therapy for people with autism spectrum disorder. Cochrane Database Syst Rev. (2014) 2014:Cd004381. doi: 10.1002/14651858.CD004381 24936966 PMC6956617

[21] Reschke-Hernández AE . History of music therapy treatment interventions for children with autism. J Music Ther. (2011) 48:169–207. doi: 10.1093/jmt/48.2.169 21938891

[22] Ghasemtabar SN Hosseini M Fayyaz I Arab S Naghashian H Poudineh Z . Music therapy: An effective approach in improving social skills of children with autism. Adv BioMed Res. (2015) 4:157. doi: 10.4103/2277-9175.161584 26380242 PMC4550953

[23] Bieleninik L Geretsegger M Mössler K . Effects of Improvisational Music Therapy vs Enhanced Standard Care on Symptom Severity Among Children With Autism Spectrum Disorder: the TIME-A Randomized Clinical Trial (vol 318, pg 525, 2017). Jama-Journal Am Med Assoc. (2021) 325:1473–3. doi: 10.1001/jama.2021.4108 PMC5817481 28787504

[24] Geretsegger M Fusar-Poli L Elefant C Mössler KA Vitale G Gold C . Music therapy for autistic people. Cochrane Database Syst Rev. (2022) 5:CD004381. doi: 10.1002/14651858.CD004381 PMC9082683 35532041

[25] Ke X Song W Yang M Li J Liu W . Effectiveness of music therapy in children with autism spectrum disorder: A systematic review and meta-analysis. Front Psychiatry. (2022) 13:905113. doi: 10.3389/fpsyt.2022.905113 36276324 PMC9582596

[26] Page MJ McKenzie JE Bossuyt PM Boutron I Hoffmann TC Mulrow CD . The PRISMA 2020 statement: an updated guideline for reporting systematic reviews. BMJ. (2021) 29:372–n71. doi: 10.1136/bmj.n71 PMC8005924 33782057

[27] American Psychiatric Association . Diagnostic and Statistical Manual of Mental Disorders. 5th (DSM-5) edition. Arlington (VA: American Psychiatric Publishing (2013).

[28] World Health Organization . ICD-11: international statistical classification of diseases 11th revision, in: The Global Standard for Diagnostic Health Information. Available online at: icd.who.int/en (Accessed 17 December 2024).

[29] Sterne JA Savović J Page MJ Elbers RG Blencowe NS Boutron I . RoB 2: a revised tool for assessing risk of bias in randomised trials. BMJ. (2019) 366:l4898. doi: 10.1136/bmj.l4898 31462531

[30] Cohen J Cohen P West SG Aiken LS . Applied multiple regression/correlation analysis for the behavioral sciences. New York:Routledge (2013).

[31] Chandler J Cumpston M Li T Page MJ Welch V . Cochrane handbook for systematic reviews of interventions Vol. 10. Hoboken: Wiley (2019). p. ED000142. doi: 10.1002/14651858.ED000142 PMC10284251 31643080

[32] Higgins JP Thompson SG Deeks JJ Altman DG . Measuring inconsistency in meta-analyses. BMJ. (2003) 327:557–60. doi: 10.1136/bmj.327.7414.557 PMC192859 12958120

[33] DerSimonian R Laird N . Meta-analysis in clinical trials revisited. Contemp Clin Trials. (2015) 45:139–45. doi: 10.1016/j.cct.2015.09.002 PMC4639420 26343745

[34] Egger M Smith GD Schneider M Minder C . Bias in meta-analysis detected by a simple, graphical test. BMJ. (1997) 315:629–34. doi: 10.1136/bmj.315.7109.629 PMC2127453 9310563

[35] Shi L Lin L . The trim-and-fill method for publication bias: practical guidelines and recommendations based on a large database of meta-analyses. Medicine. (2019) 98:e15987. doi: 10.1097/md.0000000000015987 31169736 PMC6571372

[36] Zhang L Liu CL Long YJ Zhao SF Huang YM Liu CY . Effects of Orff music therapy combined with repetitive transcranial magnetic stimulation in the treatment of children with autism spectrum disorder. Chin Med Herald. (2023) 20:86–9. doi: 10.20047/j.issn1673-7210.2023.05.20

[37] Rao JM Feng SM Ji L . Influence of TCM five-element music on psychological behavior and music development of children with autism. J Mudanjiang Med College. (2023) 44:78–81. doi: 10.13799/j.cnki.mdjyxyxb.2023.06.040

[38] Yurteri N Akdemir M . The effect of music therapy on autistic symptoms and quality of life in children with autism spectrum disorder. Anadolu Psikiyatri Dergisi. (2019) 20:436–41. doi: 10.5455/apd.12505

[39] Zhou Y Liu Y Wang CN . Clinical observation of ultrasound combined with five elements of music in the treatment of autism spectrum disorder. Chin Traditional Med Modern Distance Education. (2023) 21:128–30. doi: 10.3969/j.issn.1672-2779.2023.04.047

[40] Zhao YH Wen CB Qi YM Li En . Clinical observation of music therapy in the treatment of children with autism. J Pract Chin Med Internal Med. (2020) 34:23–6. doi: 10.13729/j.issn.1671-7813.z20200029. SN.

[41] Fan QL Ding MY Cheng W Su LS Zhang YP Liu QX . The clinical effects of Orff music therapy on children with autism spectrum disorder: a comprehensive evaluation. Front Neurology. (2024) 15:1387060. doi: 10.3389/fneur.2024.1387060 PMC11188925 38903168

[42] Zhou YH . Influence of music therapy combined with speech training on the recovery of speech function in children with autism. Chin convalescent Med. (2018) 27:1157–9. doi: 10.13517/j.cnki.ccm.2018.11.013

[43] Shen S Kong JL Yang J . Observation on the effect of guided education combined with parent-child cooperative music therapy on autistic children. Chin Health Care · Acad edition. (2022) 40:172–5.

[44] He YS Liu GH Zhang YH Xie NM Lin JL Hu RF . A prospective randomized controlled study of the effects of parent-child collaborative music therapy on children with autism spectrum disorder and their mothers. Chin J Contemp Pediatrics. (2022) 24:472–81. doi: 10.7499/j.issn.1008-8830.2201105 PMC9154363 35644186

[45] Li N ZL LI Zhao Y Liu ZH Wang T Zhao YP . Clinical effect of Songbo music therapy on children with autism spectrum disorder. Massage Rehabil Med. (2021) 12:34–7. doi: 10.19787/j.issn.1008-1879.2021.21.010

[46] Xiao Q Fang W . Effects of auditory integration training and rehabilitation combined with music therapy on language recovery and social ability of autistic children. Contemp Nurse · Compr edition. (2023) 30:103–6. doi: 10.19791/j.cnki.1006-6411.2023.07.032

[47] Brondino N Fusar-Poli L Rocchetti M Provenzani U Barale F Politi P . Complementary and alternative therapies for autism spectrum disorder. Evid Based Complement Alternat Med. (2015), 258589. doi: 10.1155/2015/258589 26064157 PMC4439475

[48] LaGasse AB . Social outcomes in children with autism spectrum disorder: a review of music therapy outcomes. Patient Related Outcome Measures. (2017) 8:23–32. doi: 10.2147/prom.S106267 28260959 PMC5325134

[49] Kemper KJ Danhauer SC . Music as therapy. South Med J. (2005) 98:282–8. doi: 10.1097/01.SMJ.0000154773.11986.39 15813154

[50] Shi Z Wang S Chen M Hu A Long Q Lee Y . The effect of music therapy on language communication and social skills in children with autism spectrum disorder: a systematic review and meta-analysis. Front Psychol. (2024) 15:1336421. doi: 10.3389/fpsyg.2024.1336421 38774719 PMC11106491

[51] Gotham K J Risi S Pickles A Lord C . The autism diagnostic observation schedule: revised algorithms for improved diagnostic validity. J Autism Dev Dis. (2007) 37:613–27. doi: 10.1007/s10803-006-0280-1 17180459

[52] Zentner M Eerola T . Rhythmic engagement with music in infancy. Proc Natl Acad Sci U.S.A. (2010) 107:5768–73. doi: 10.1073/pnas.1000121107 PMC2851927 20231438

[53] Redondo Pedregal C Heaton P . Autism, music and Alexithymia: A musical intervention to enhance emotion recognition in adolescents with ASD. Res Dev Disabil. (2021) 116:104040. doi: 10.1016/j.ridd.2021.104040 34329821

[54] Shi Z Lin G Xie Q . Meta-analysis of the effects of music therapy on the emotional, verbal, behavioral, and social skills of children with autism. Nurs Res. (2016) 30:10. doi: 10.3969/j.issn.1009-6493.2016.08.009

