# Supplementary material for: The effectiveness of music therapy in improving behavioral symptoms among children with autism spectrum disorders: a systematic review and meta-analysis
Source: Front Psychiatry. 2025 Jan 14;15:1511920. doi: 10.3389/fpsyt.2024.1511920 (PMC11783185; doi:10.3389/fpsyt.2024.1511920)
Supplement: Supplementary file 1 [file Supplementaryfile1.docx]

Table S1 **The comprehensive search tactics**

We searched the following databases with no language or publication restrictions:

SinoMed: https://www.sinomed.ac.cn/

Cochrane Library: http://www-cochranelibrary-com-443.ca.ilibs.cn/

Web of Science: https://webofscience.clarivate.cn/wos/alldb/basic-search

PubMed: https://pubmed.ncbi.nlm.nih.gov/

Embase: https://www-embase-com.uic.sjlib.cn/

exemplify a Cochrane search procedure

Cochrane Library September 18, 2024

[Cochrane Central Register of Controlled Trials](https://www.cochranelibrary.com/)

Search Strategy:

| **#** | **Searches** | **Results** |
| --- | --- | --- |
| 1 | MeSH descriptor: [Autistic Disorder] explode all trees | 1624 |
| 2 | “autistic disorder” or “autism spectrum disorders” or “early infantile autism” or autistic or autism or “autistic traits” or ASD | 6605 |
| 3 | MeSH descriptor: [Music Therapy] explode all trees | 1290 |
| 4 | MeSH descriptor: [Music] explode all trees | 1234 |
| 5 | #1 or #2 | 6605 |
| 6 | #3 or #4 | 2218 |
| 7 | music or “music intervention” or “music therapy” or music* or improvis* or “music training” | 8619 |
| 8 | #6 or #7 | 8619 |
| 9 | #5 and #8 | 162 |

Figure S2 **Meta-regression analysis of publication year, sample size, music style, intervention type, measurement tools, and intervention duration on ASD behavioral symptoms**


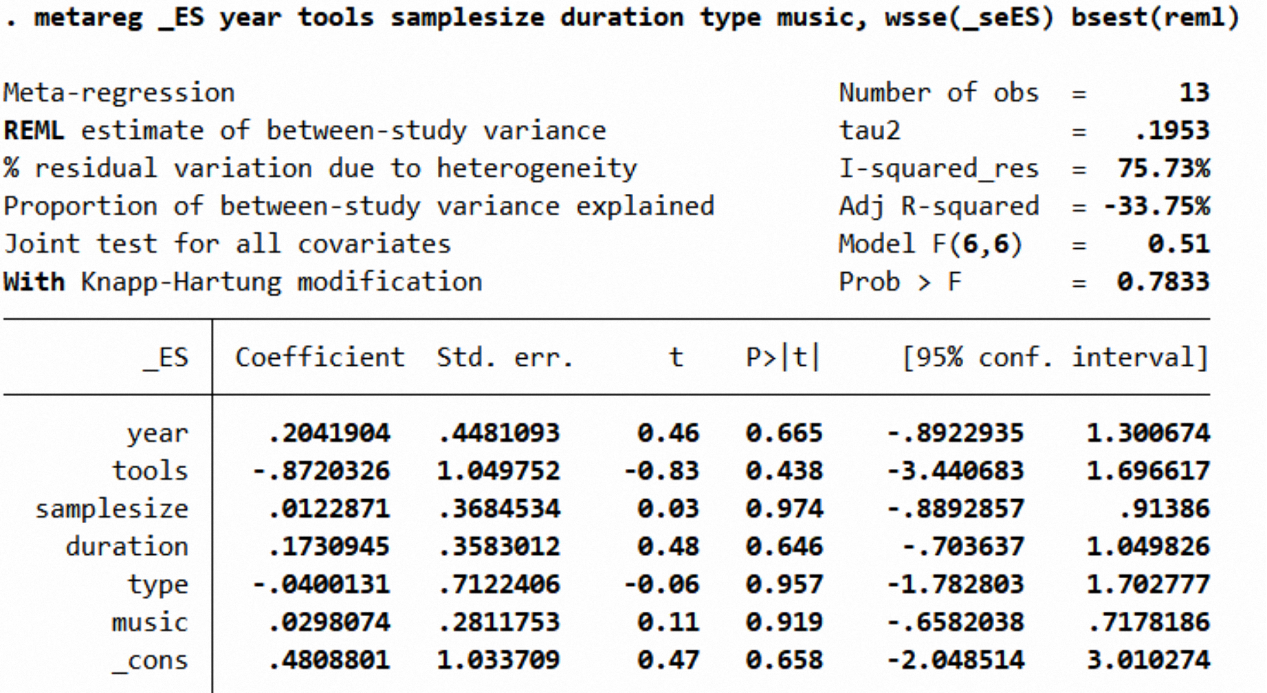


Figure S3 **Subgroup analysis of intervention duration (S3A), and publication year (S3B) on ASD behavioral symptoms**


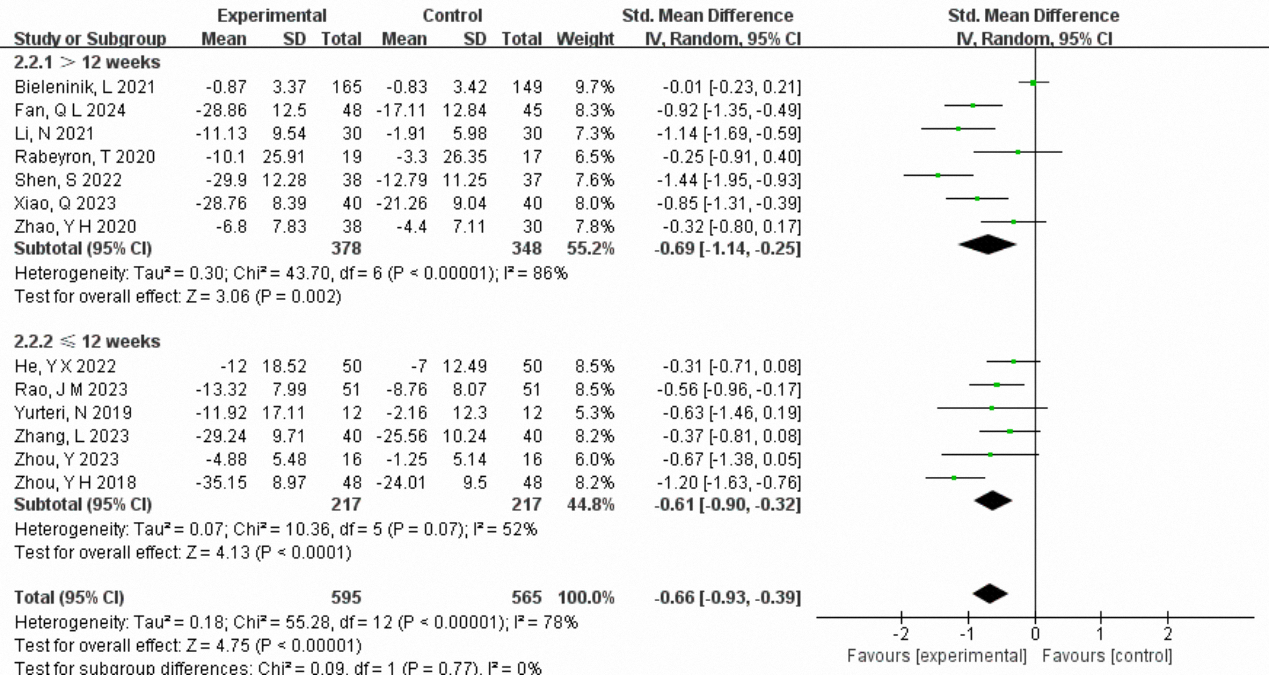


**Figure S3A**


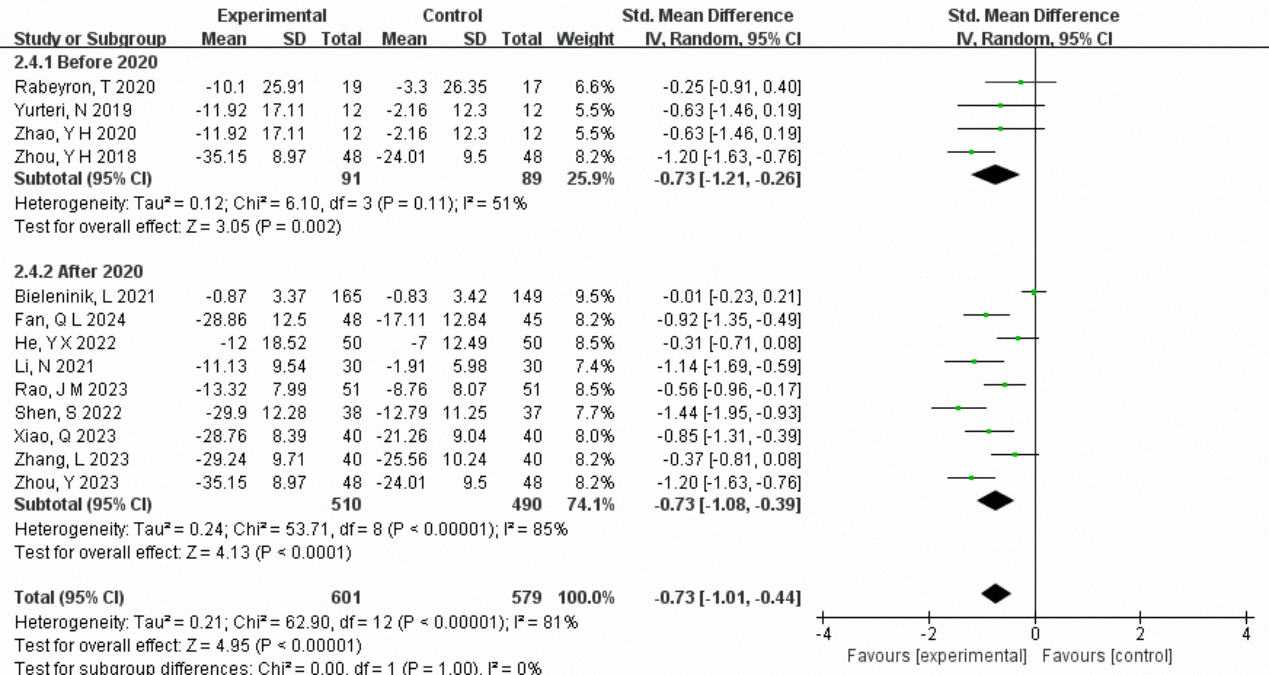


**Figure S3B**

Figure S4 **Test of sensitivity analysis on ASD behavioral symptoms**

**
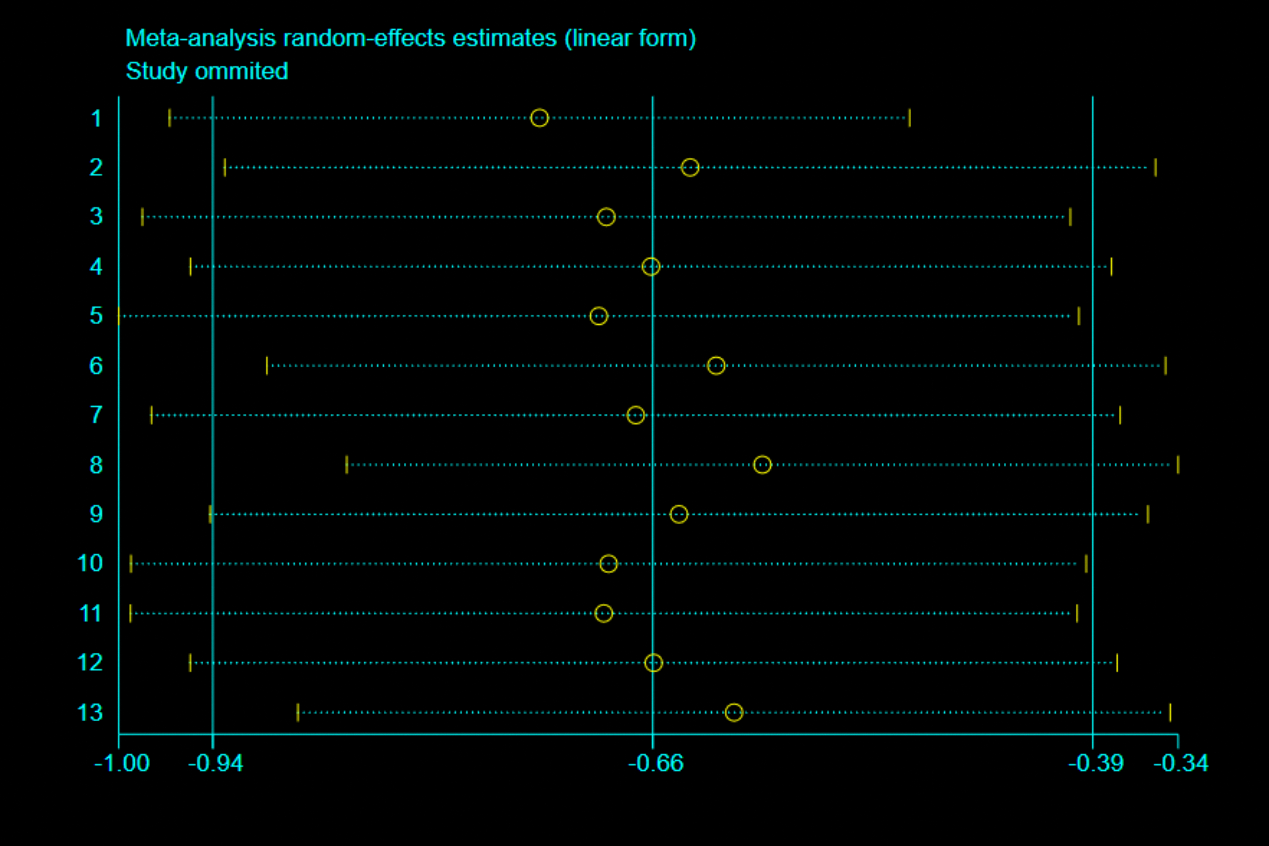
**

Figure S5 **Test of publication bias (S5A: funnel plot, S5B: Egger’s tests, S5C: trim-and-fill) on ASD behavioral symptoms**

**
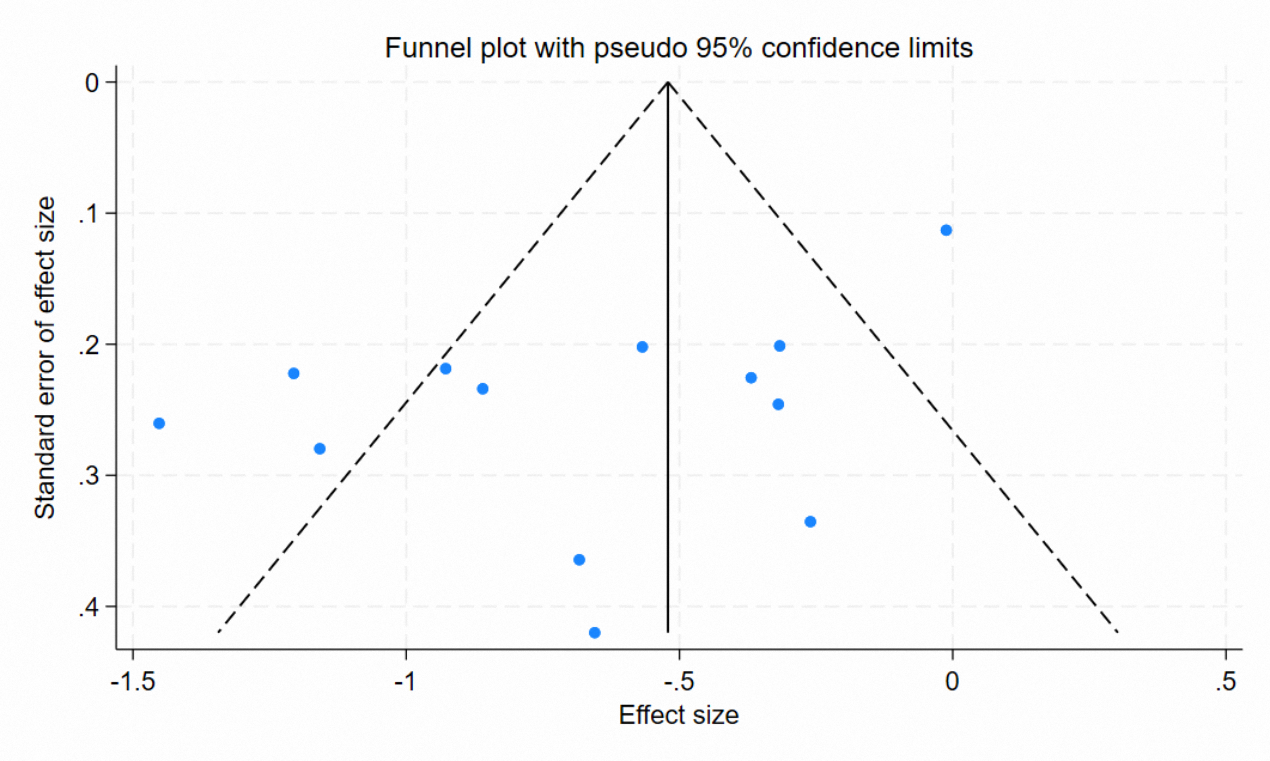
**

**Figure S5A**

**
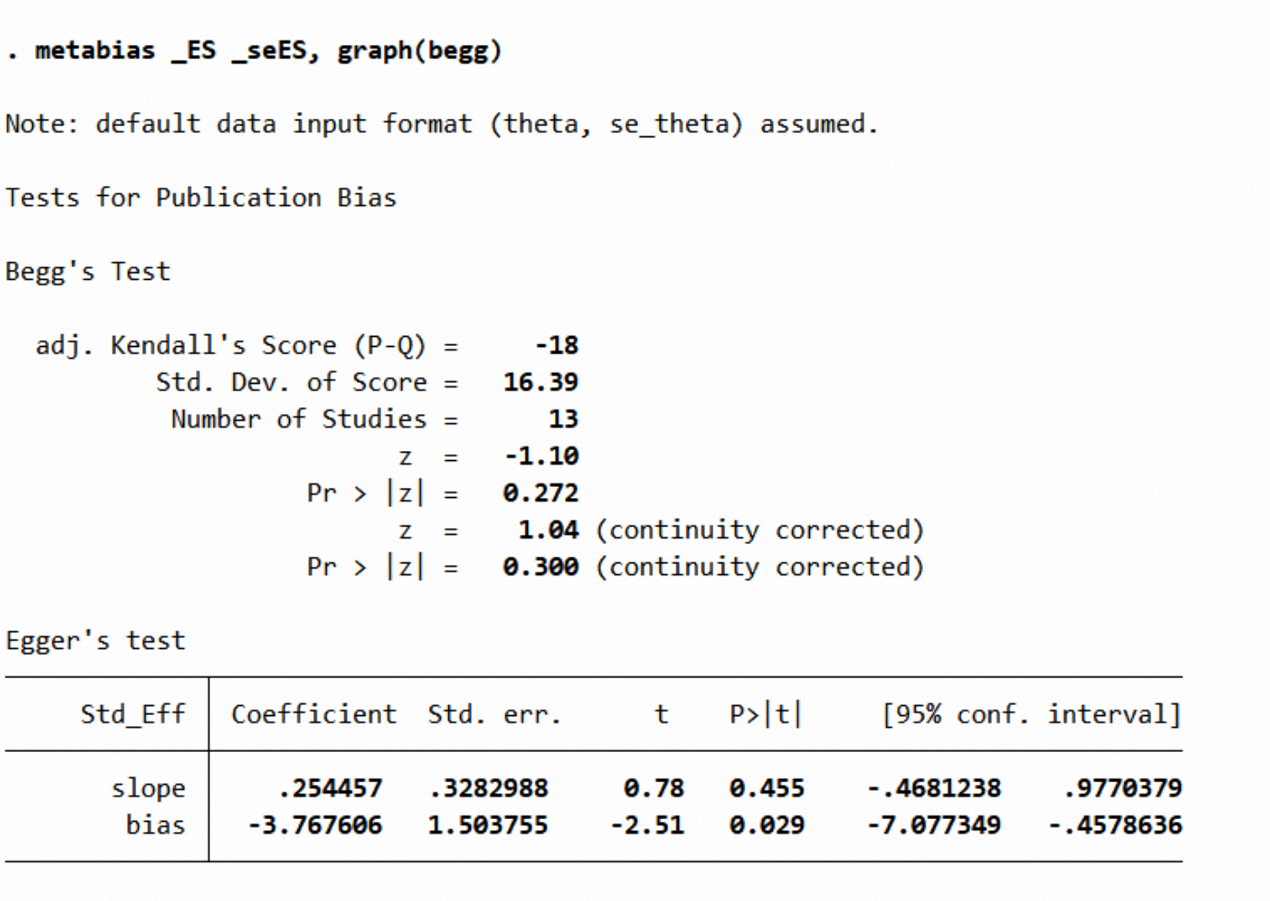
**

**Figure S5B**

**
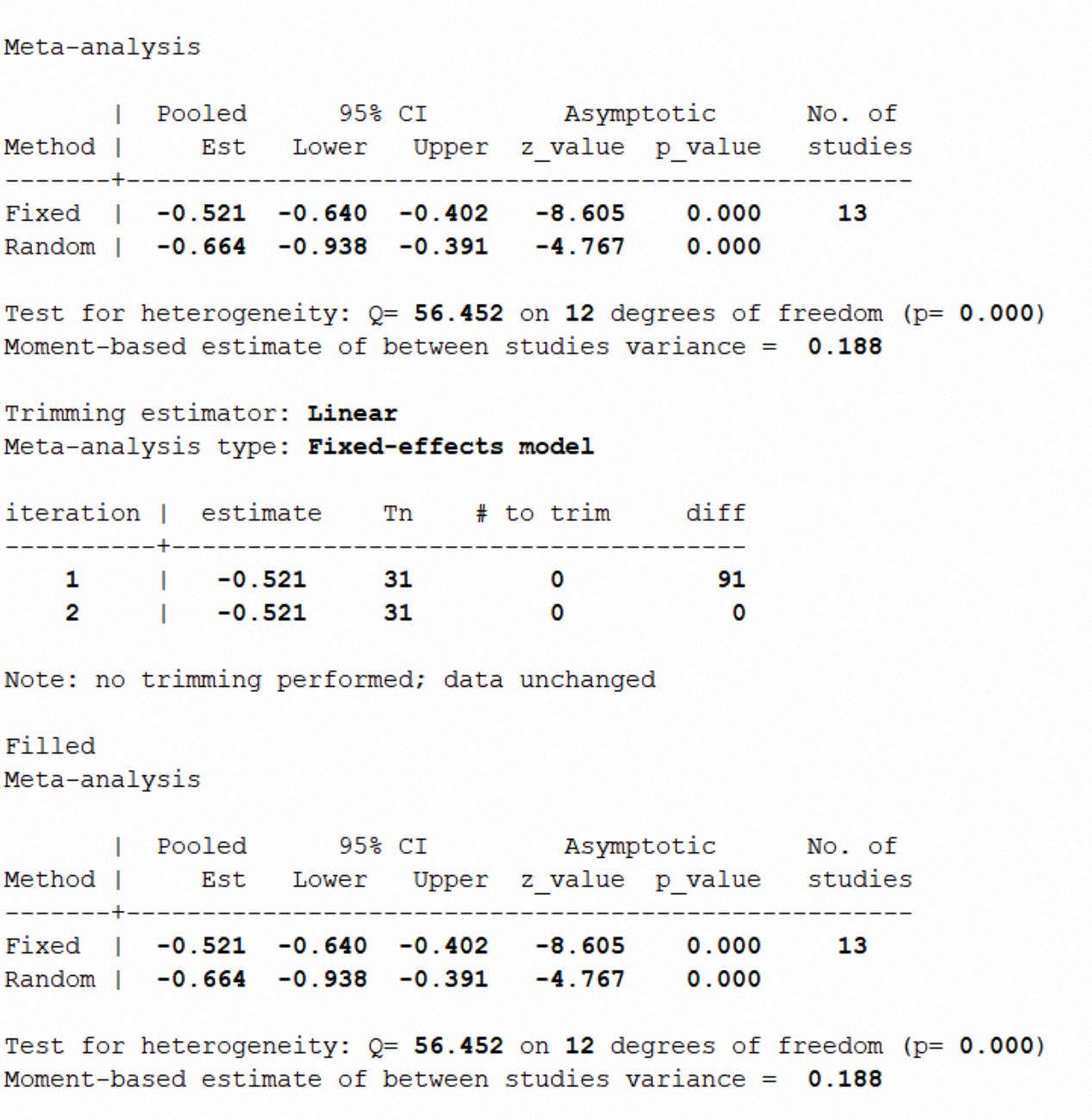
**

**Figure S5C**
